# Radiation-Associated Angiosarcoma as a Presentation of Disease Progression in a Patient on Immunotherapy for Metastatic Non-small Cell Lung Cancer

**DOI:** 10.7759/cureus.44724

**Published:** 2023-09-05

**Authors:** Hannah Tan, Beatrice E Torere, Sherry Okun, Benjamin Hinton

**Affiliations:** 1 Hematology and Oncology, East Tennessee State University, Johnson City, USA; 2 Internal Medicine, North Mississippi Medical Center, Tupelo, USA; 3 Pathology, North Mississippi Medical Center, Tupelo, USA; 4 Radiation Oncology, North Mississippi Medical Center, Tupelo, USA

**Keywords:** radiation-associated cancer, secondary primary cancer, nccn guidelines, stage iv nsclc, metastatic non-small cell lung cancer, adenocarcinoma of the lung, non-small cell lung carcinoma (nsclc), epithelioid angiosarcoma

## Abstract

Non-small cell lung cancer (NSCLC) is the dominant form of lung cancer, comprising around 85% of cases. Stage 4 NSCLC has a grim prognosis; however, immunotherapy and radiation therapy have become vital treatments for advanced-stage NSCLC, despite the risk of inducing a second primary malignancy.

This case report focuses on a 45-year-old female diagnosed with NSCLC and metastasis to the 11th thoracic vertebral body. After various treatments, including radiation, a potential radiation-associated secondary malignancy, epithelial angiosarcoma, was discovered. Following treatment modification, the patient achieved complete metabolic remission, highlighting the importance of clinicians being cautious about secondary primary cancers in NSCLC patients with a history of radiation therapy. Accurate diagnosis through biopsy and continuous surveillance are essential in managing NSCLC patients effectively.

## Introduction

The prognosis for stage 4 non-small cell lung cancer (NSCLC), a malignancy from lung epithelial cells, has notably improved in recent years. The five-year overall survival rate increased from 7% before 2015 to 23.2%, thanks to the approval of immune checkpoint inhibitors (ICIs) like nivolumab and pembrolizumab. ICIs, which enhance the body's immune response against cancer cells, serve as the first line of treatment, either alone or in combination with chemotherapy [1]. Radiation therapy is also a vital treatment option for many patients with advanced NSCLC, used either palliatively in stage 4 or curatively in earlier stages. Increased life expectancy in stage 4 NSCLC due to these advanced therapeutics may lead to a higher incidence of radiation-associated complications, including secondary malignancies [2]. Radiation-associated angiosarcoma, a rare and aggressive vascular malignancy, typically occurs in patients post-radiation treatment, most frequently in breast cancer cases. The incidence is significantly lower in NSCLC due to the shorter survival time of patients with locally advanced NSCLC compared to early-stage breast cancer patients. Secondary sarcomas generally occur years after radiation therapy with a median of seven years [3]. Given the nonspecific radiological findings, a high level of clinical suspicion and a tissue biopsy are necessary for a definitive diagnosis [2]. Due to its rarity, treatment strategies for radiation-associated angiosarcoma are not well-defined. Surgical removal, chemotherapy, radiation, or ICIs are reported options, but the prognosis remains bleak for non-surgical angiosarcoma, with survival expectancy under six months [3].

In advanced NSCLC, initial diagnosis requires tissue biopsy and molecular studies for biomarker-guided targeted therapy. These involve evaluating molecular markers, such as the epidermal growth factor receptor (EGFR), to guide decisions regarding targeted tyrosine kinase inhibitors (TKIs) [4]. However, in general practice, it is not mandatory to repeat the biopsy for patients who progressed after many lines of treatment, including chemotherapy and immunotherapy [5]. Post-immunotherapy treatment options for advanced NSCLC are limited, and the overall survival benefit of additional chemotherapy with or without anti-angiogenesis is uncertain. Previous studies report that only a small percentage of eligible patients receive systemic chemotherapy after failing ICIs and that this additional chemotherapy does not significantly impact overall survival [2,6].

This report discusses a patient who underwent extensive treatments, including two chemotherapy regimens, three radiation treatment plans at separate times, and immunotherapy. Six to seven years after her first radiation for her stage 4 NSCLC, a computed tomography (CT) scan indicated disease progression. Given her treatment history and the elapsed time since radiation therapy, a re-biopsy was performed, suspecting a possible radiation-associated secondary malignancy. The biopsy confirmed this suspicion, leading to a revised treatment strategy, adding paclitaxel to her existing pembrolizumab regimen [7].

Remarkably, the patient achieved a complete metabolic response, as demonstrated by a subsequent positron emission tomography (PET) scan. This response indicates that the continued administration of ICIs alongside chemotherapy may have an additive curative effect, especially in patients with secondary malignancy post radiation therapy. This case underlines the importance of re-biopsy and modification of treatment plans in patients with advanced NSCLC, particularly those showing disease progression after initial treatment, to consider the possibility of secondary malignancies and their respective treatment options [7,8].

## Case presentation

**Figure 1:**
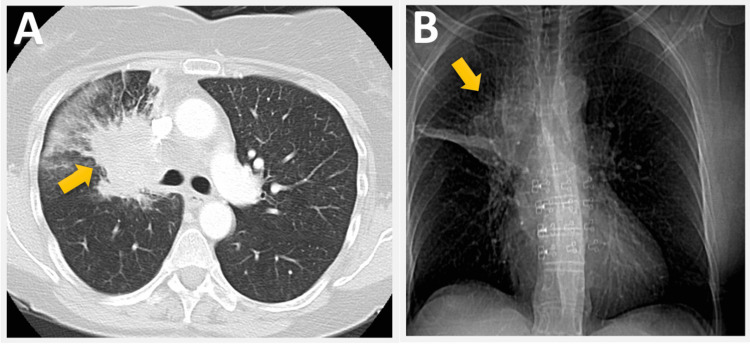
CT scan of the chest demonstrating a large spiculated 7.5 x 6 cm mass in the right upper lobe (arrow in A) and extensive mediastinal lymphadenopathy (arrow in B).

A 45-year-old female patient presented with swallowing difficulties and unintentional weight loss of approximately 20 lbs over three months. She was a former smoker with a 20-pack-year history who had quit smoking a few months before the presentation. An esophagogastroduodenoscopy (EGD) revealed normal results, while a chest CT scan revealed a large spiculated 7.5 x 6 cm mass in the right upper lobe and extensive mediastinal lymphadenopathy (Figure 1).

**Figure 2:**
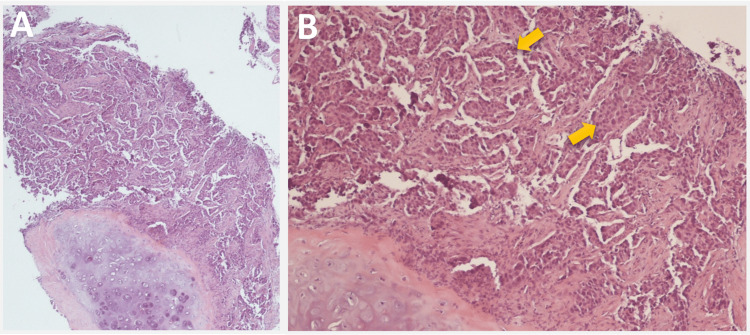
Histopathological images with hematoxylin and eosin stain from right upper lobe biopsy at 4x (A) and 10x (B) magnification showing invasive moderately differentiated papillary adenocarcinoma cells (arrows in B), indicative of primary non-small cell lung cancer.

Two days later, the patient underwent a bronchoscopy with transbronchial fine needle aspiration (FNA) of the right upper lobe mass. Biopsy confirmed the diagnosis of invasive moderately differentiated papillary adenocarcinoma, pointing to a lung primary (Figure 2).

**Figure 3:**
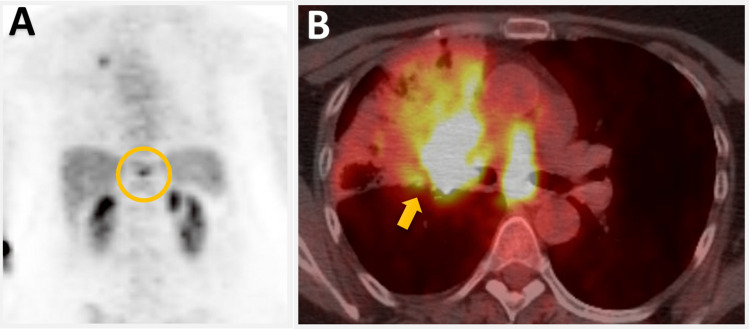
PET scan demonstrates intense hypermetabolic activity in the T11 vertebral body suggestive of potential metastasis to this region (circle in A) and original right lobe mass (arrow in B).

A subsequent PET scan suggested potential metastasis to the 11th thoracic vertebral body (Figure 3).

**Figure 4:**
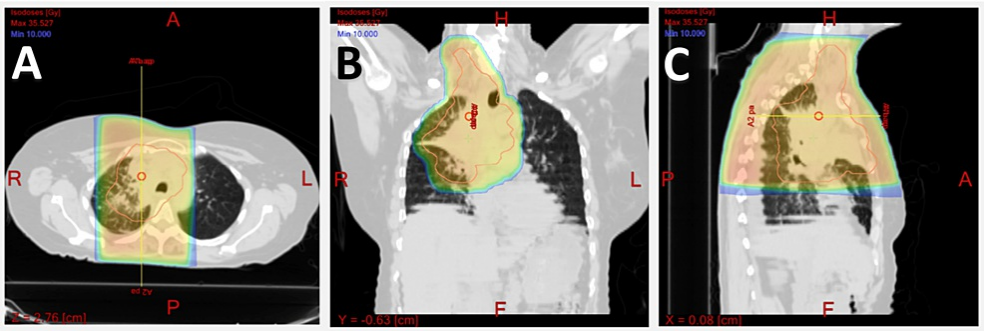
CT scans of the target radiation field with a color wash display of the dose (at a minimum of 10 Gy) for a 30 Gy plan showing the dose distribution in the single-isocenter plan. Gross tumor volume is outlined in red. A = transverse; B = frontal; C = sagittal.

However, molecular tests revealed no targetable mutations. Following a discussion about the potential benefits of clinical trial chemotherapy and nivolumab, the patient sought a second opinion from another facility. Upon her return to our oncology clinic, she opted for first-line conventional therapy with carboplatin and pemetrexed, prior to the immunotherapy era. Shortly after starting chemotherapy, she developed fever and chills due to obstructive pneumonia, which necessitated two weeks of 30 Gy (Gray) radiation delivered in 10 fractions with a daily dose of 3 Gy by three-dimensional conformal radiation therapy (3DCRT) to the right upper lobe mass (Figure 4). She also received amoxicillin and clavulanic acid for seven days.

**Figure 5:**
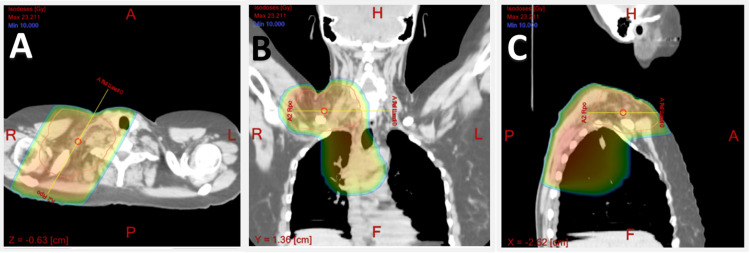
CT scans of the target radiation field with a color wash display of the dose (at a minimum of 10 Gy) for a 20 Gy plan showing the dose distribution in the single-isocenter plan. Gross tumor volume is outlined in red. A = transverse; B = frontal; C = sagittal.

Two months post-radiation, the patient reported escalating neck pain and a right neck mass. A CT scan showed an increased size and number of lymph nodes in the right supraclavicular region and right anterior and posterior cervical chains, signaling disease progression. She underwent a two-week course of 20 Gy radiation delivered in 10 fractions with a daily dose of 3 Gy by 3DCRT (Figure 5), and then treatment with nivolumab was started a month later, achieving disease control for three years.

**Figure 6:**
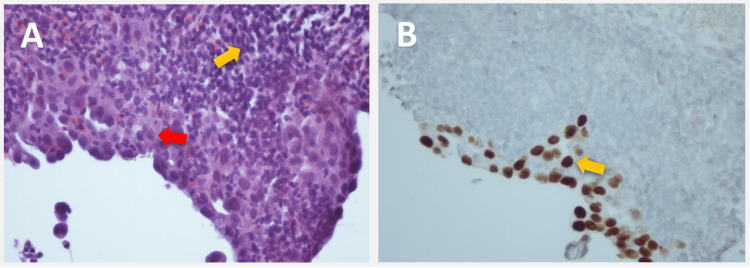
Histopathological images from right neck lymphadenopathy biopsy at 40x magnification. (A) Hematoxylin and eosin stain with benign lymphocytes (yellow arrow) and tumor cells (red arrow). (B) Positive thyroid transcription factor-1 (TTF-1) immunostaining showing findings of a few non-small cell lung cancer adenocarcinoma cells (arrow), indicating disease progression.

During nivolumab treatment, she reported worsening lymphadenopathy in her right neck. A PET scan noted mildly enlarged and hypermetabolic posterior right cervical lymph nodes, with no evidence of metastasis elsewhere. Although the first lymph node biopsy was reported as non-diagnostic, on follow-up after two months, CT scans of the chest, neck, and head showed increased size, number, and enhancement of right level five cervical lymph nodes, suggesting potential nodal metastases. A re-biopsy from right neck lymphadenopathy confirmed NSCLC disease progression (Figure 6).

**Figure 7:**
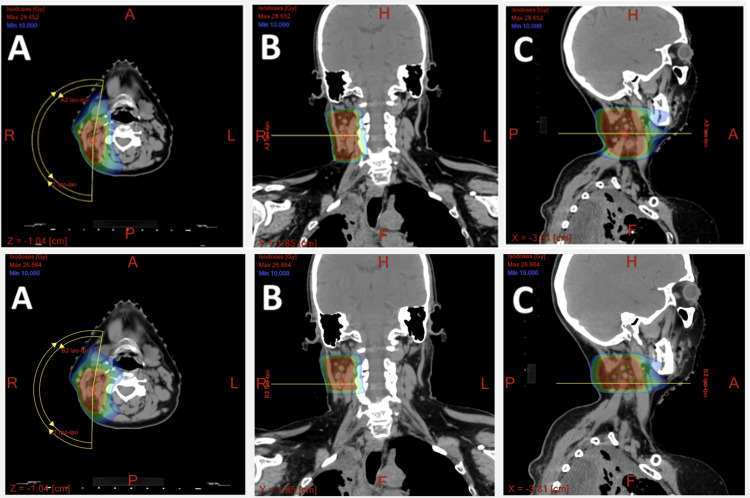
CT scans of the target radiation field with a color wash display of the dose (at a minimum of 10 Gy) for a total 50 Gy plan showing the dose distribution in the single-isocenter plan. Gross tumor volume is highlighted in red. Top row = right neck radiation. Bottom row = right neck boost radiation. A = transverse; B = frontal; C = sagittal.

Given her age, excellent performance status, and oligometastatic disease post radiation to her right neck, we intensified her treatment plan to include carboplatin and paclitaxel with concurrent radiation therapy. She received five weeks of 50 Gy radiation delivered in 25 fractions by intensity-modulated radiotherapy (IMRT) to control her right neck condition (Figure 7). Due to the high dose of radiation, IMRT was used rather than 3DCRT after discussion with the patient.

**Figure 8:**
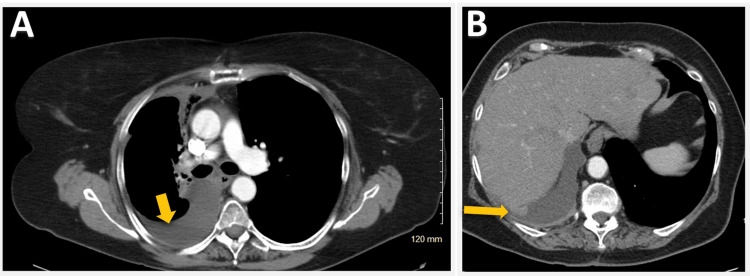
CT scan of the chest showing right lung pleural effusion (yellow arrow in A) and pleural nodularity (yellow arrow in B).

**Figure 9:**
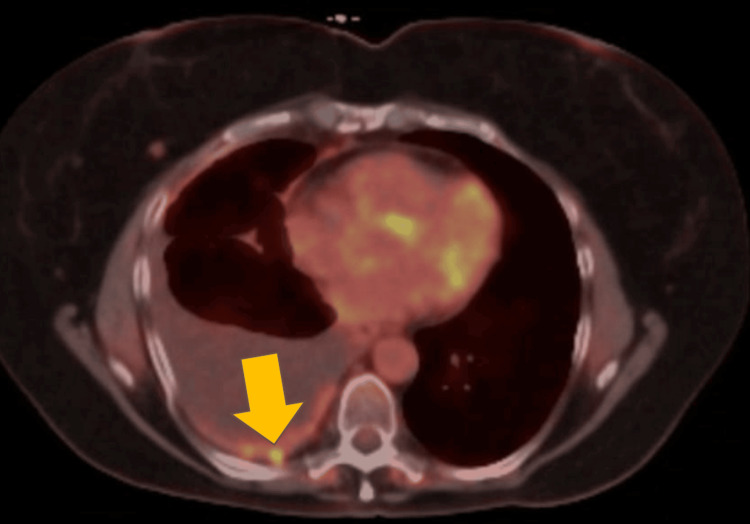
PET scan showing right basilar pleural nodularity (yellow arrow).

As pembrolizumab could be administered less frequently than nivolumab, the patient opted to switch ICIs in May 2020. The patient's condition remained stable post chemoradiation treatment for the following three years. However, a follow-up CT of the chest (Figure 8) and PET scan (Figure 9) revealed worsening right pleural effusion, enlarged pleural-based nodules at the base and apex, and lymphadenopathy at the right paratracheal, and right hilar regions, typically indicative of disease progression.

**Figure 10:**
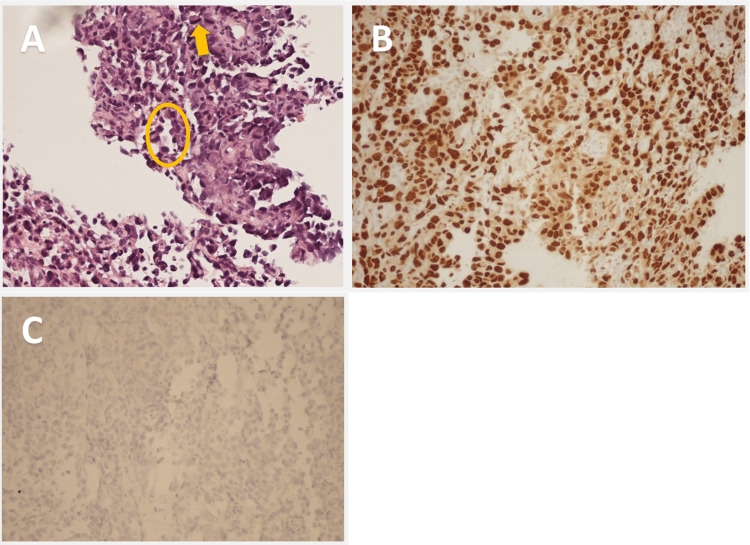
Histopathological images from right basilar pleural nodule biopsy demonstrating a second primary epithelial angiosarcoma. (A) Hematoxylin and eosin stain at 20x magnification showing tumor mitotic activity (arrow) and poorly formed vascular channels (circle) strongly indicating angiosarcoma. (B) ERG nuclear immunostaining of angiosarcoma tumor showing diffuse strong nuclear expression or positivity. Adenocarcinoma of the lung would be negative for ERG. (C) Negative thyroid transcription factor (TTF-1) immunostaining supports an angiosarcoma diagnosis.

A repeat biopsy, performed 6.5 years after her initial radiation exposure, from the right basilar pleural nodule identified epithelial angiosarcoma (Figure 10).

**Figure 11:**
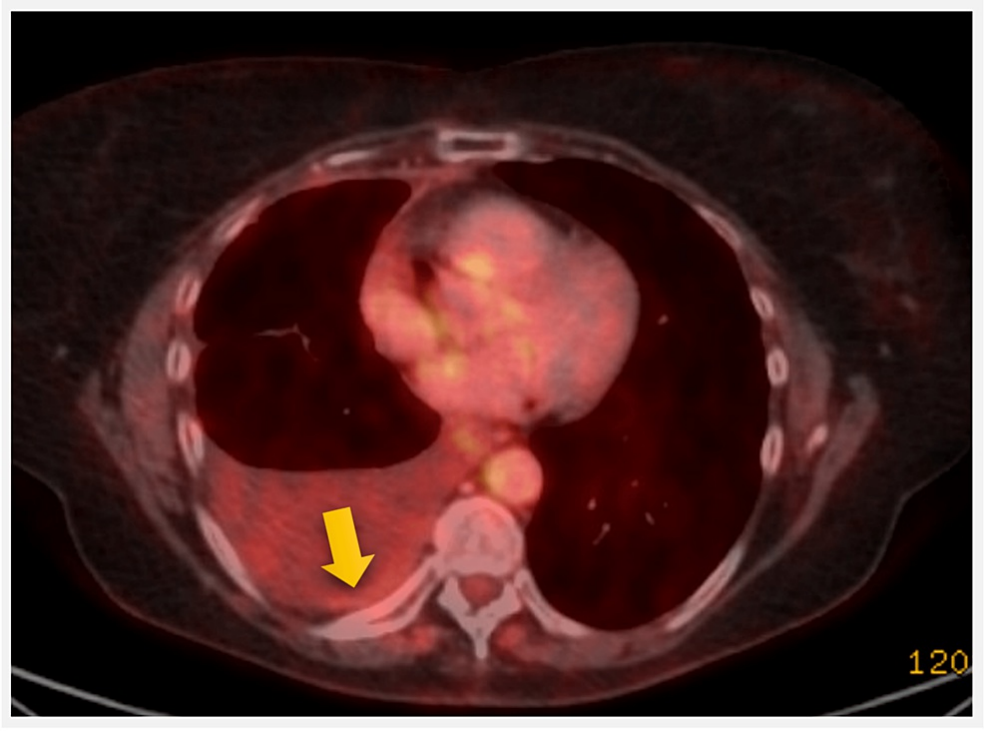
PET scan showing complete metabolic remission (arrow) from notably pleural base nodes.

Comprehensive molecular studies revealed non-targetable mutations, including RB1, BRAF D594N, and TP53, low tumor mutation burden, and stable microsatellite instability status. Consequently, she initiated weekly paclitaxel treatment. After four weeks, a follow-up PET scan displayed a complete metabolic response from her pleural base nodules and other sites (Figure 11).

## Discussion

The advent of ICIs has dramatically reshaped the treatment paradigm for NSCLC. However, most lung cancer patients eventually experience disease progression during immunotherapy. Resistance to immunotherapy may occur at various stages, manifesting as either primary or secondary resistance [9]. Oligometastatic progression is a frequent pattern of disease progression following secondary resistance to immunotherapy. In patients with advanced NSCLC, local radiotherapy for oligometastatic lesions, combined with continued ICI treatment, has demonstrated promising results, resulting in prolonged progression-free survival and overall survival [10].

Our patient developed a progression of oligometastatic lesions in the presented case, confirmed to be the same type of NSCLC through biopsy. Given her young age and satisfactory performance, she underwent curative-dose radiation and concurrent chemotherapy. After this treatment, she continued ICI maintenance therapy and achieved an additional three years of progression-free survival after her initial disease progression from ICI therapy.

The occurrence of a second primary tumor is common after the diagnosis of a primary tumor and treatment with ICIs. While some studies suggest that immunotherapy may decrease the incidence of second primary tumors, others report an increased risk of second primary cancer post immunotherapy [11,12]. The hypothesis is that the immune effect stimulated by ICIs is systemic and may offer protection against secondary carcinogenesis. However, in this case, the efficacy of immunotherapy for NSCLC did not prevent the development of a second primary tumor, which was the cause of disease progression identified in the imaging report.

In clinical practice, tumor progression is often the initial consideration when new lesions or enlarging masses are reported during treatment, prompting recommendations for switching treatment regimens [13]. Had a biopsy not been performed in this case, the subsequent treatment step for presumed NSCLC progression would likely have been to cease pembrolizumab and initiate docetaxel, with or without an anti-angiogenesis agent [14]. However, since a biopsy confirmed the presence of epithelial angiosarcoma while the patient was on pembrolizumab, selecting a more specific systemic chemotherapy regimen for epithelial angiosarcoma was reasonable, despite the lack of level I evidence-based recommendations. Expert opinion from the National Comprehensive Cancer Network (level III) suggests paclitaxel, anthracycline, or other options for treating epithelial angiosarcoma [15]. As the patient's stage 4 NSCLC remained well-controlled with pembrolizumab, the decision was made to continue pembrolizumab for the stage 4 NSCLC and add paclitaxel for the epithelial angiosarcoma. Following four weeks of weekly paclitaxel with pembrolizumab, the patient's disease progression was reassessed via a PET scan. Complete metabolic remission was observed after adding four weeks of weekly paclitaxel to the treatment regimen. Although immunotherapy alone did not prevent the development of epithelial angiosarcoma in this case, continuing pembrolizumab may have offered clinical benefits in managing the epithelial angiosarcoma [7,8]. This strategy provides the patient hope for potential durable responses from ICIs in treating angiosarcoma.

## Conclusions

This case underscores the critical role of repeat biopsy in patients facing cancer progression despite undergoing multiple lines of treatment, including radiation. The remarkable outcome of the index patient exemplifies how a repeat biopsy not only resulted in the diagnosis of an additional primary cancer but also played a vital role in guiding her treatment decisions and facilitating her recovery. The patient's prior history of radiation treatments within the past seven years heightened suspicions of a potential radiation-associated separate cancer. This case emphasizes the need for personalized treatment approaches. It motivates further investigation into the combined use of ICIs and chemotherapy for patients with radiation-associated epithelial angiosarcoma or other angiosarcomas.
